# Effects of l-Arginine Plus Vitamin C Supplementation on l-Arginine Metabolism in Adults with Long COVID: Secondary Analysis of a Randomized Clinical Trial

**DOI:** 10.3390/ijms24065078

**Published:** 2023-03-07

**Authors:** Riccardo Calvani, Jacopo Gervasoni, Anna Picca, Francesca Ciciarello, Vincenzo Galluzzo, Hélio José Coelho-Júnior, Clara Di Mario, Elisa Gremese, Sara Lomuscio, Anna Maria Paglionico, Lavinia Santucci, Barbara Tolusso, Andrea Urbani, Federico Marini, Emanuele Marzetti, Francesco Landi, Matteo Tosato

**Affiliations:** 1 Fondazione Policlinico Universitario A. Gemelli IRCCS, 00168 Rome, Italy; 2 Department of Medicine and Surgery, LUM University, 70010 Casamassima, Italy; 3 Università Cattolica del Sacro Cuore, 00168 Rome, Italy; 4 Immunology Core Facility, Gemelli Science Technological Park (GSTeP), Fondazione Policlinico Universitario A. Gemelli IRCCS, 00168 Rome, Italy; 5 Metabolomics Research Core Facility, Gemelli Science and Technology Park (GSTeP), Fondazione Policlinico Universitario A. Gemelli IRCCS, 00168 Rome, Italy; 6 Department of Chemistry, Sapienza University of Rome, 00185 Rome, Italy

**Keywords:** post-acute COVID-19 syndrome, SARS-CoV-2, ADMA, flow-mediated dilation, nitric oxide, nutraceuticals, oral supplement, persistent symptoms, endothelial dysfunction, metabolomics

## Abstract

Altered l-arginine metabolism has been described in patients with COVID-19 and has been associated with immune and vascular dysfunction. In the present investigation, we determined the serum concentrations of l-arginine, citrulline, ornithine, monomethyl-l-arginine (MMA), and symmetric and asymmetric dimethylarginine (SDMA, ADMA) in adults with long COVID at baseline and after 28-days of l-arginine plus vitamin C or placebo supplementation enrolled in a randomized clinical trial, compared with a group of adults without previous history of SARS-CoV-2-infection. l-arginine-derived markers of nitric oxide (NO) bioavailability (i.e., l-arginine/ADMA, l-arginine/citrulline+ornithine, and l-arginine/ornithine) were also assayed. Partial least squares discriminant analysis (PLS–DA) models were built to characterize systemic l-arginine metabolism and assess the effects of the supplementation. PLS–DA allowed discrimination of participants with long COVID from healthy controls with 80.2 ± 3.0% accuracy. Lower markers of NO bioavailability were found in participants with long COVID. After 28 days of l-arginine plus vitamin C supplementation, serum l-arginine concentrations and l-arginine/ADMA increased significantly compared with placebo. This supplement may therefore be proposed as a remedy to increase NO bioavailability in people with long COVID.

## 1. Introduction

l-arginine metabolism is involved in the regulation of several biological processes, including immune and vascular function [1,2]. Two main metabolic pathways are associated with the pleiotropic activities of l-arginine: its conversion to nitric oxide (NO) by NO synthase (NOS) or l-arginine catabolism to ornithine by arginase [3,4]. NOS and arginase entertain reciprocal regulatory interactions that impact NO bioavailability [5]. NO is a master regulator of cardiovascular function, metabolism, neurotransmission, and immunity [6]. The flux of l-arginine towards NO synthesis is associated with beneficial effects on immune and vascular health [5]. On the other hand, upregulation of arginase inhibits NO production and promotes immune and endothelial dysfunction [4]. Several clinical conditions, including hypertension, diabetes, and inflammatory diseases, are characterized by the rewiring of l-arginine metabolism towards increased arginase activity [7,8,9]. In addition to the NOS/arginase dyad, some l-arginine derivatives can modulate NO bioavailability. Methylarginine moieties, including monomethyl-l-arginine (MMA) and symmetric and asymmetric dimethylarginine (SDMA and ADMA), are released following proteolysis of arginine-methylated proteins [10]. In vitro and in vivo data have shown that MMA, SDMA, and ADMA may inhibit NOS [11]. Indeed, elevated circulating levels of ADMA and SDMA have been identified as independent risk factors for cardiovascular events and all-cause mortality across different populations [12,13,14].

Perturbations in l-arginine metabolism have been described in patients with COVID-19 across all disease stages [5,15,16]. During acute COVID-19, higher arginase activity shifts l-arginine away from NO synthesis to induce immune dysregulation and endothelial dysfunction, which both increase the risk of thrombosis, arterial stiffening, and vascular occlusion [5]. A low l-arginine-to-ornithine ratio, indicative of upregulated arginase activity, has been found in patients with COVID-19 and children with multisystem inflammatory syndrome (MIS-C), and has been associated with reduced circulating levels of l-arginine compared with healthy controls [17]. Arginine shortage and enhanced arginase activity have also been associated with expansion of myeloid-derived suppressor cells, lymphopenia, and lymphocyte dysfunction in COVID-19 patients with severe acute respiratory distress syndrome (ARDS) [18,19]. Moreover, elevated serum levels of ADMA and SDMA at hospital admission were associated with disease severity [20] and predicted in-hospital mortality in patients with COVID-19 [21]. Eight months after acute infection, young and middle-aged COVID-19 survivors showed reduced serum levels of l-arginine compared with controls without evidence of previous SARS-CoV-2 infection [16]. 

Based on these observations, interventions targeting l-arginine metabolism have been proposed to increase NO bioavailability and contrast immune and vascular complications of COVID-19 [1,5,22]. In vitro, l-arginine supplementation restored the proliferative capacity of T-cells obtained from COVID-19 patients with ARDS [19]. Oral l-arginine supplementation reduced the need for oxygen therapy and the length of hospital stay in patients with severe COVID-19 [23]. The combination of l-arginine plus vitamin C, which may support NOS activity in endothelial cells [24], showed a synergistic antiviral action on SARS-CoV-2 in vitro by inhibiting its main protease M^pro^ [25]. A supplement containing l-arginine and vitamin C relieved the burden of persistent symptoms and improved perceived exertion in a large cohort of adults with long COVID [26]. The latter condition is diagnosed in individuals who, at three months of an acute symptomatic COVID-19 episode, have persistence of symptoms for at least two months, which cannot be explained by any other possible diagnosis [27]. Finally, we recently showed that a 28-day oral supplementation with l-arginine plus vitamin C restored circulating l-arginine levels and improved walking performance, muscle strength, endothelial function, and fatigue in adults with long COVID [16].

In the present investigation, we conducted secondary analyses of a randomized clinical trial that involved 28 days of supplementation with l-arginine plus vitamin C or placebo. Data from a group of adults without previous history of SARS-CoV-2-infection were also analyzed. For this study, we assayed a comprehensive panel of metabolites pertaining to l-arginine metabolism to obtain further insights into systemic arginine metabolism and NO bioavailability in adults with long COVID. We also assessed the effects of l-arginine plus vitamin C supplementation on l-arginine metabolites. 

## 2. Results

### 2.1. Characteristics of Study Population and Concentrations of l-Arginine Metabolites at Baseline

Fifty-seven participants were included in the present investigation: 46 adults with long COVID (47.8 ± 9.4 years; 65.2% women) enrolled in a randomized clinical trial [16] and a group of 11 age- and sex-matched controls without evidence of previous SARS-CoV-2 infection (48.8 ± 11.1 years; 55.5% women). As previously reported [16], approximately half of those with long COVID needed hospitalization during the acute COVID-19 episode, and four (8.7%) were admitted to an intensive care unit. The average time elapsed from COVID-19 diagnosis to the inclusion in the study was 252.6 ± 113.7 days. Participants with long COVID had been randomized to receive either the l-arginine plus vitamin C (*n* = 23) or the placebo (*n* = 23) intervention for 28 days [16]. Demographic and anthropometric characteristics of study participants, standard blood biochemistry, as well as concentrations of l-arginine metabolites at baseline are reported in Table 1.

### 2.2. l-Arginine Metabolism in Participants with Long COVID and Controls

To gain insights into possible differences in l-arginine metabolism between participants with long COVID and healthy controls, baseline serum concentrations of l-arginine-related analytes were processed through partial least squares discriminant analysis (PLS–DA). To obtain an unbiased validation of the results and estimate the confidence intervals (CIs) of the main figures of merit, repeated double cross-validation (rDCV) was conducted with 10 and eight cancelation groups in the outer and inner loops, respectively, and permutation tests with 1000 randomizations.

The optimal model complexity was found to be 3 ± 1 latent variables and yielded an average classification accuracy of 80.2 ± 3.0%, corresponding to 78.1 ± 3.2% and 88.9 ± 5.2% correct classification rate for participants with long COVID and controls, respectively. The non-parametric estimation of the distribution of these figures of merit under the null hypothesis by permutation testing indicated that they were statistically significant (*p* < 0.001).

**Figure 1:**
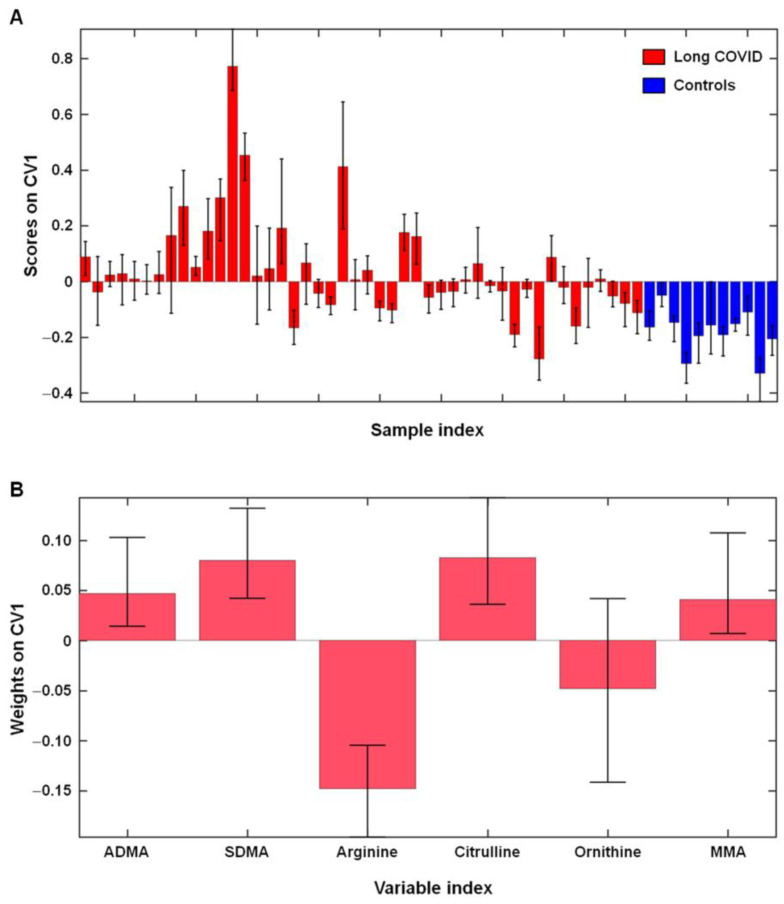
Outer loop sample scores (**A**) and variable weights (**B**) along the only canonical variate of the partial least squares discriminant analysis model depicting differences in l-arginine metabolism between participants with long COVID (*n* = 46) and healthy controls (*n* = 11). Abbreviations: ADMA, asymmetric dimethylarginine; MMA, monomethyl-l-arginine; SDMA, symmetric dimethylarginine.

A graphical representation of the discriminant ability of the model is depicted in Figure 1A. The figure shows the rDCV outer loop sample scores and the variable weights along the only canonical variate of the model.

Inspection of the contribution of individual l-arginine metabolites to the classification model indicated that five out of the six measured analytes contributed significantly to the discrimination: ADMA, MMA, SDMA, citrulline (on average higher in participants with long COVID), and l-arginine (on average higher in controls) (Figure 1B).

According to reference values proposed by major clinical medical laboratories [28,29,30], serum levels of ADMA and SDMA in participants with long COVID may be suggestive of endothelial dysfunction and increased cardiovascular risk.

In participants with long COVID, lower circulating levels of l-arginine led to reduced l-arginine/ADMA (mean difference: −152.7, 95% CI: −196.58 to −106.66; effect size = 0.81; *p* < 0.0001), lower global arginine bioavailability ratio (GABR) (mean difference: −0.73, 95% CI: −1.03 to −0.45; effect size = 0.82; *p* < 0.0001), and lower l-arginine-to-ornithine ratio (mean difference: −0.99, 95% CI: −1.45 to −0.57; effect size = 0.76; *p* < 0.0001) than healthy controls. These findings indicate that l-arginine bioavailability is reduced and, therein, NO biosynthetic capacity may be impaired in participants with long COVID compared with those without evidence of previous SARS-CoV-2 infection. 

### 2.3. Effects of l-Arginine Plus Vitamin C Supplementation on l-Arginine Metabolism in Participants with Long COVID

A PLS–DA model was built to explore the effects of 28-day supplementation with l-arginine plus vitamin C or placebo on l-arginine metabolism in participants with long COVID. To account for the repeated measures design of the study, the classification model was built using, for each participant, the difference between values at 28 days and those at baseline (Table 2).

**Figure 2:**
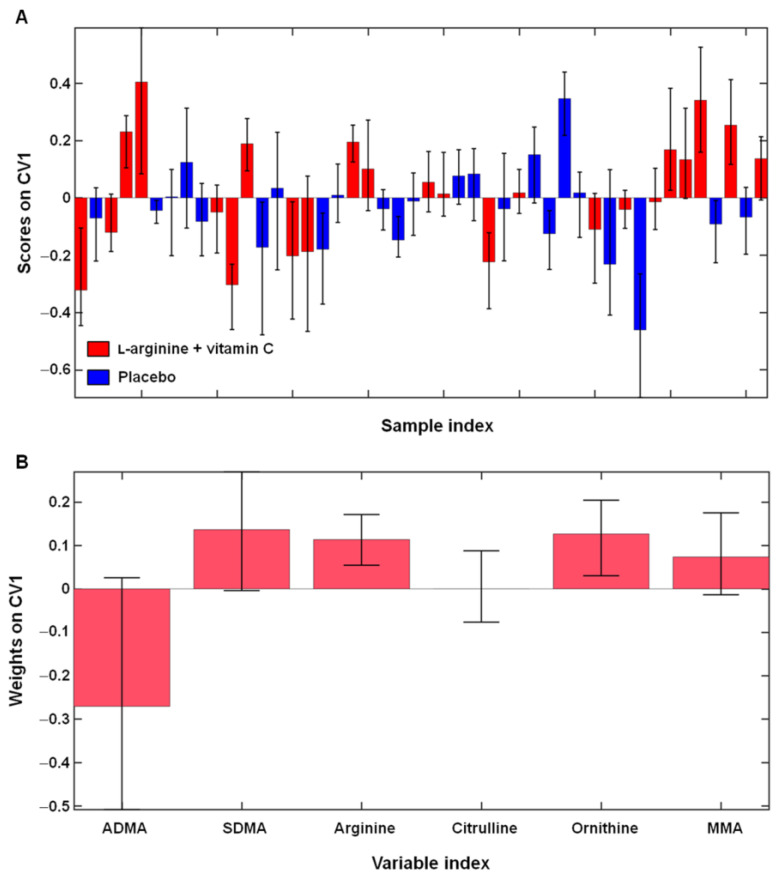
Outer loop sample scores (**A**) and variable weights (**B**) along the only canonical variate (CV) of the partial least squares discriminant analysis model assessing differences in l-arginine metabolism after 28 days of l-arginine plus vitamin C supplementation (*n* = 23) compared with placebo (*n* = 23). Abbreviations: ADMA, asymmetric dimethylarginine; MMA, monomethyl-l-arginine; SDMA, symmetric dimethylarginine.

The optimal model complexity was found to be 4 ± 1 latent variables. The model had low discrimination power, with an average classification accuracy of 58.5 ± 5.8%, corresponding to 54.3 ± 6.3% and 62.6 ± 8.6% of correct classification in participants who received l-arginine plus vitamin C and those in the placebo group, respectively (Figure 2A).

As previously reported [16], l-arginine plus vitamin C supplementation induced a significantly greater increase in circulating l-arginine levels compared with placebo (mean difference: 62.4 µM, 95% CI: 11.1 to 113.7 µM; effect size = 0.72; *p* = 0.02). This finding was confirmed by the inspection of the variable weights plot of the PLS–DA model (Figure 2B). Although the increase in serum l-arginine concentrations observed in the active treatment group did not result in significant changes in GABR or l-arginine-to-ornithine ratio, the difference in l-arginine/ADMA between intervention groups was close to statistical significance (*p* = 0.05; Table 2).

**Figure 3:**
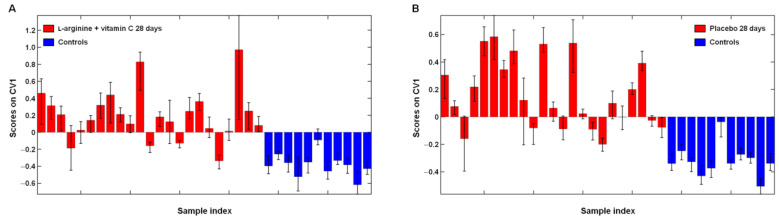

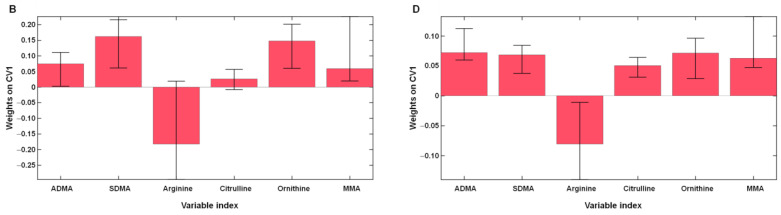
Outer loop sample scores (**A**,**B**) and variable weights (**C**,**D**) along the only canonical variate (CV) of partial least squares discriminant analysis models assessing differences in l-arginine metabolism in participants with long COVID after l-arginine plus vitamin C (*n* = 23) or placebo supplementation (*n* = 23) compared with healthy controls (*n* = 11). Abbreviation: ADMA, asymmetric dimethylarginine; MMA, monomethyl-l-arginine; SDMA, symmetric dimethylarginine.

To assess whether l-arginine plus vitamin C supplementation induced changes in l-arginine metabolism towards healthy reference values, two PLS–DA models were built, comparing data at 28 days from the active treatment group and placebo versus healthy controls (Figure 3).

**Figure 4:**
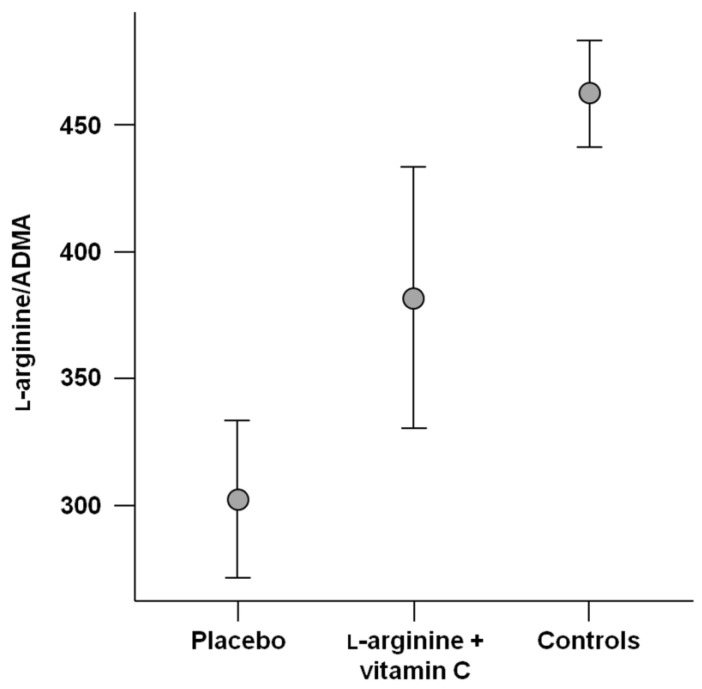
l-arginine/asymmetric dimethylarginine (ADMA) after 28 days of treatment in participants who received placebo (*n* = 23) or l-arginine plus vitamin C (*n* = 23) and in healthy controls (*n* = 11). Values are mean ± standard deviation.

Both PLS–DA models had a high mean classification accuracy (85.2 ± 2.6% and 84.5 ± 3.1%, respectively) and indicated persistent disruption of l-arginine metabolism in participants with long COVID, regardless of treatment allocation (Figure 3A,B). However, following l-arginine plus vitamin C supplementation, l-arginine and citrulline no longer contributed to the discrimination between participants with long COVID and healthy controls (Figure 3C,D). In addition, after 28 days of l-arginine plus vitamin C supplementation, mean l-arginine/ADMA values were significantly different from both those in placebo-treated participants (*p* = 0.03) and healthy controls (*p* = 0.01), with a shift towards healthy reference values (Figure 4).

## 3. Discussion

In the present investigation, we showed that l-arginine metabolism was altered in a group of adults with long COVID eight months after the diagnosis of COVID-19 compared with age- and sex-matched adults without history of SARS-CoV-2 infection. l-arginine metabolism was still disrupted after 28-day supplementation with l-arginine plus vitamin C. However, in those who received the active intervention, both serum l-arginine concentrations and l-arginine/ADMA, a marker of NO biosynthetic capacity, significantly shifted towards healthy reference values compared with participants who were allocated to placebo.

Accumulating evidence indicates that arginine metabolism is altered in COVID-19 patients [17,19,31,32,33]. In particular, low circulating l-arginine levels and upregulated arginase activity have been associated with reduced NO bioavailability and immune and vascular dysfunction in acute COVID-19 [15]. Low arginine-to-ornithine ratio and low GABR, as well as two-fold increase in circulating levels of ADMA, were found in severely ill COVID-19 patients [34]. This suggests that SARS-CoV-2 infection may induce endothelial dysfunction and a pro-thrombotic vascular phenotype acting both on NOS substrate availability and enzyme activity. Our findings show that similar perturbations in l-arginine metabolism may be found in adults with long COVID several months after the acute episode. Notably, low GABR was associated with the development of coronary artery disease and increased risk of major adverse cardiovascular events over a 3-year follow-up in a cohort of 1010 patients undergoing elective cardiac catheterization [35]. In addition, reduced GABR and l-arginine-to-ornithine ratio were associated with markers of endothelial dysfunction and increased risk of cardiovascular mortality in patients referred for coronary angiography [36]. Increased circulating levels of ADMA were found in conditions characterized by impaired NO synthesis and endothelial dysfunction, such as hypertension, diabetes, atherosclerosis, and cerebrovascular diseases [37,38,39,40,41,42,43]. Elevated ADMA levels increase the risk of recurrent cardiovascular events or death in patients with a history of acute coronary disease [44,45], unstable angina [46], or diabetes [47]. Low l-arginine/ADMA is an independent risk factor for atherosclerosis [48] and microangiopathy-related cerebral damage [49], and has shown to be a better predictor of all-cause mortality than ADMA alone [48,50]. In this scenario, the reduced indices of l-arginine/NO bioavailability found in adults with long COVID-19 in the present investigation suggest a role for altered l-arginine metabolism in increasing the risk of endotheliopathy and long-term cardiovascular events [51,52,53,54].

l-arginine plus vitamin C supplementation increased circulating levels of l-arginine and shifted l-arginine/ADMA values towards healthy reference. Owing to its arginine-like structure, ADMA may directly compete with l-arginine both for its transport into the cell via the cationic amino acid transporter and NOS binding [55,56]. It follows that NO bioavailability may be influenced by the balance between l-arginine and ADMA [1]. Low l-arginine/ADMA results in a net inhibition of NO production [57]. Oral l-arginine supplementation may re-equilibrate l-arginine/ADMA, increase NO synthesis, and improve endothelial function [1,58]. Our previous findings corroborate this hypothesis, since in adults with long COVID supplemented with l-arginine plus vitamin C, the increase in circulating l-arginine concentrations and l-arginine/ADMA was associated with a significant improvement in flow-mediated dilation (FMD), a measure of NO-dependent endothelial reactivity, compared with placebo [16]. These results are in line with those from a meta-analysis of randomized clinical trials showing that short-term oral l-arginine supplementation improved endothelial function in individuals with reduced FMD [59]. In this context, l-arginine supplementation may be particularly suited for people with ascertained endothelial dysfunction and low l-arginine/ADMA, such as those with long COVID, since l-arginine supplementation in individuals with high FMD and low ADMA levels (or normal l-arginine/ADMA ratio) failed to improve either NO bioavailability or endothelial function [59,60,61].

Some limitations should be considered in the interpretation of the study results. Due to the small number of participants and the single-center nature of the study, our results should be considered preliminary. Further investigation with larger populations, conducted in multiple centers, and using different study methodologies (e.g., longer intervention, crossover design) is warranted to confirm our findings. The levels of physical activity as well as dietary habits of study participants may have influenced the concentration of l-arginine metabolites and the effects of interventions. However, participants were requested to refrain from exercising, limit the ingestion of foods rich in arginine, and taking substances with vasoactive properties for at least 12 h before study visits. Due to the heterogeneity of data on vaccination status (e.g., timing, types of vaccine, number of doses, refusal to disclose vaccination status), this information was not accounted for in the analyses. The panel of metabolites assessed in the present investigation provided relevant information on differences in l-arginine metabolism between adults with long COVID and healthy controls and allowed for evaluating the effectiveness of the tested intervention. However, we cannot exclude that a more comprehensive evaluation of NO metabolism (e.g., measurement of circulating levels of nitrite, nitrate, and NO derivatives), as well as the assessment of inflammatory, vascular, or neurological markers may provide further insights into the mechanisms by which l-arginine plus vitamin C supplementation affects the outcomes of interest. Vitamin C levels were not quantified; thus, the relationship between circulating vitamin C concentrations and study outcomes could not be explored. Because l-arginine levels were not measured days after the end of the intervention, it was not possible to appreciate the duration of the beneficial effects of l-arginine plus vitamin C supplementation on the parameters of interest. Finally, it cannot be ruled out that the co-administration of other nutraceuticals may convey additional beneficial effects on l-arginine metabolism and long COVID symptoms [22,62,63]. For instance, vitamin D may have positive effects on both NO synthesis and endothelial function [64]. Vitamin D deficiency is frequent in COVID-19 survivors and is associated with poor physical performance [65]. The combined use of l-arginine, coenzyme Q10, and vitamin D was found to reduce oxidative stress and stimulate NO synthesis in cardiac and endothelial cells to a greater extent than any of those compounds alone [66]. The combination has therefore been proposed as a cardiovascular protective remedy [66]. Further studies are needed to assess whether supplementation with different combinations of nutrients may be proposed as a remedy to restore l-arginine metabolism and limit post-acute COVID-19 sequelae.

## 4. Materials and Methods

### 4.1. Study Design and Participants

Participants involved in the present investigation were adults with long COVID who were enrolled in a placebo-controlled randomized clinical trial that tested the effects of a combination of l-arginine plus vitamin C on physical performance, endothelial function, and persistent fatigue (NCT04947488) [16]. Eleven age- and sex-matched blood donors without evidence of previous SARS-CoV-2 infection were recruited and analyzed as a “healthy” reference. Trial operations were conducted at the post-acute COVID-19 outpatient clinic of the Fondazione Policlinico A. Gemelli IRCCS (Rome, Italy) from 1 July 2021 to 30 April 2022 [67]. Details on the clinical trial protocol and inclusion/exclusion criteria have been reported elsewhere [16]. Briefly, trial participants were men and women aged 20 to 60 years with a previous confirmed SARS-CoV-2 infection (certified by a positive RT–PCR molecular swab test), a negative COVID-19 test at least four weeks prior to enrolment, long COVID diagnosis according to national and international criteria [27,68], and persistent fatigue, defined as the response “most or all the time” to item seven of the Center for Epidemiological Studies Depression Scale (“I felt that everything I did was an effort”) [69]. The main exclusion criteria were: intolerance to preparations containing l-arginine or vitamin C, conditions and/or treatments that might affect trial outcomes or procedures (e.g., pregnancy or nursing, diabetes, and use of antihypertensive medications, corticosteroids, or non-steroidal anti-inflammatory drugs, immunosuppressants, nitrates), and participation in other long COVID intervention trials. Eligible participants were randomized to receive twice-daily an oral supplementation with either a combination of 1.66 g l-arginine plus 500 mg liposomal vitamin C (Bioarginina^®^ C, Farmaceutici Damor, Naples, Italy) or placebo for 28 days. The dose was selected based on previous evidence of the beneficial effects of l-arginine supplementation during acute COVID-19 [23], and on the trial methodology followed by Rizzo et al. [26] to assess effectiveness of l-arginine plus vitamin C on improving long COVID symptoms. All trial procedures were conducted in accordance with the guidelines of the International Council for Harmonisation of Technical Requirements for Pharmaceuticals for Human Use Good Clinical Practice and the principles of the Declaration of Helsinki. All participants provided written informed consent prior to enrolment.

Trial participants and healthy controls were asked to refrain from exercising and consuming any product with vasoactive properties (e.g., tobacco, caffeinated drinks) for at least 12 h before blood drawing.

### 4.2. l-Arginine Metabolism Assessment

Blood samples were collected after overnight fasting using standard collection tubes. Samples were left at room temperature for 30 min and were then centrifuged at 1000× *g* for 10 min at 4 °C. Serum aliquots were stored at −80 °C until analysis. Serum samples from participants with long COVID were collected at baseline and after 28 days of intervention. Blood samples from healthy controls were collected and processed according to the same protocol. The concentrations of l-arginine, citrulline, ornithine, ADMA, MMA, and SDMA were measured using an in-house validated liquid chromatography with tandem mass spectrometry method [70]. The chromatographic separation was performed with an ACQUITY UPLC I-Class System (Waters, Milford, MA, USA) using a HILIC column. Analyte detection was performed using a triple quadrupole Xevo-TQs Micro (Waters) equipped with an electrospray ion source operating in positive ion mode. A multiple reaction monitoring experiment was optimized for the detection and quantification of l-arginine and its metabolites.

l-arginine-derived indexes associated with endothelial and immune dysfunction, such as l-arginine/ADMA, GABR (l-arginine/ornithine+citrulline), and l-arginine-to-ornithine ratio, were assessed in all study participants across the different timepoints [17,35,48].

### 4.3. Statistical Analysis

Personal characteristics of study participants are reported as mean ± standard deviation or median (interquartile range) for continuous variables, and as absolute values (percentages) for categorical variables. Normal distribution of data was assessed via the Shapiro–Wilk test. Changes from baseline for continuous variables are expressed as deltas (i.e., values at 28 days minus values at baseline) and differences between groups were evaluated using Student’s *t*-test for normally distributed variables or Mann–Whitney U test for skewed variables. Mean differences and effect size values (Cohen’s d for Student’s *t*-test and rank biserial correlation for Mann–Whitney U) were reported. One-way analysis of variance and post hoc tests were used to compare mean concentration values of l-arginine metabolites between participants with long COVID and healthy controls. All tests were two-sided with statistical significance set at *p* < 0.05. All analyses were performed using Jamovi freeware version 2.0.0.0 (The Jamovi project, 2021; https://www.jamovi.org, accessed on 27 February 2023).

Multivariate classification models, based on PLS–DA [71], were built to gain a more comprehensive insight into l-arginine metabolism during post-acute COVID-19 and to assess the effects of l-arginine plus vitamin C supplementation in adults with long COVID. 

PLS–DA is a classification method that exploits the advantages of the PLS algorithm for dealing with correlated variables. The PLS algorithm was originally developed for regression problems and relies on the projection of the predictor matrix, X, onto a reduced space of orthogonal latent variables, yielding a matrix of scores, T (coordinates of the samples onto the latent variables subspace):

T = XR (1)

R being a matrix of weights determining the projection.

A regression model is then established between the scores T and the response, y, to be predicted:

y = Tq (2)

q being the regression coefficients.

The same approach can be used for classification by using a dummy binary y coding for class belonging: the elements of y can be either 1 if the sample belongs to a category or 0 if it belongs to the other category. 

First, classification models were built to evaluate differences in l-arginine metabolism between long COVID participants (y = 1) and healthy controls (y = 0). Then, the effects of l-arginine plus vitamin C supplementation on systemic l-arginine metabolism were tested building models to discriminate between active treatment (y = 1) and placebo (y = 0). Finally, PLS–DA models were built to explore whether l-arginine supplementation could revert l-arginine metabolic profiles of long COVID participants towards the healthy reference status (using either l-arginine plus vitamin C or placebo-treated participants as one category (y = 1) and healthy controls as the other (y = 0)). 

Model validation was achieved through rDCV [72]. rDCV consists of two loops of cross-validation nested into one another: the outer loop mimics an external test set, while the inner loop is used for model selection (i.e., choosing the optimal number of latent variables). The procedure was repeated 50 times, changing the distribution of samples in the different cancelation groups, which allowed CIs to be calculated for all model parameters and figures of merit.

To account for the repeated-measure design of the intervention study, the classification models were built using, for each participant, the difference between values at 28 days and values at baseline. Analyses were performed using in-house routines running under MATLAB R2015b environment (The MathWorks, Natick, MA, USA).

## 5. Conclusions

In the present investigation, we showed that perturbations in l-arginine metabolism indicative of reduced l-arginine/NO bioavailability were found in adults with long COVID at eight months from acute disease compared with controls without previous history of SARS-CoV-2 infection. After 28-day supplementation with l-arginine plus vitamin C, serum l-arginine levels and l-arginine/ADMA ratio, a marker of NO biosynthetic capacity, increased relative to placebo. Given the preliminary nature of our findings, further studies are needed to conclusively establish whether l-arginine plus vitamin C supplementation restores l-arginine metabolism in adults with long COVID.

## Figures and Tables

**Table 1:** Characteristics of Study Participants and Concentrations of l-Arginine Metabolites at Baseline.

Characteristic	Long COVID		Healthy Controls (*n* = 11)
	l-Arginine + Vitamin C (*n* = 23)	Placebo (*n* = 23)	
Age, years	47.3 ± 10.7	48.4 ± 8.0	48.8 ± 11.1
Women, *n* (%)	15 (65.2)	15 (65.2)	6 (55.5)
BMI, kg/m^2^	25.6 ± 5.6	25.6 ± 4.0	25.8 ± 4.7
Glucose, mmol/L	4.8 ± 0.6	4.8 ± 0.6	5.0 ± 0.3
Total Cholesterol, mmol/L	5.4 ± 1.1	5.3 ± 1.1	4.6 ± 0.5
Albumin, mmol/L	0.66 ± 0.05	0.65 ± 0.04	0.67 ± 0.05
Creatinine, µmol/L	69.7 ± 16.4	68.2 ± 14.9	64.0 ± 11.7
CRP, nmol/L, median (IQR)	33.3 (80.9)	33.3 (22.9)	33.1 (18.4)
Hemoglobin, g/L	14.3 ± 1.5	14.2 ± 1.4	14.3 ± 1.2
White Blood Cells, 10^9^ L	5.6 ± 2.0	6.1 ± 1.8	5.8 ± 2.0
l-arginine, µM	192.7 ± 74.1	196.6 ± 80.6	221.6 ± 31.3
Citrulline, µM	41.4 ± 13.2	41.6 ± 11.9	30.1 ± 7.5
Ornithine, µM	122.5 ± 43.6	124.9 ± 56.6	82.9 ± 12.5
ADMA, µM	0.60 ± 0.14	0.64 ± 0.19	0.48 ± 0.02
MMA, µM	0.13 ± 0.05	0.14 ± 0.06	0.10 ± 0.02
SDMA, µM	0.71 ± 0.15	0.77 ± 0.25	0.53 ± 0.11
Arginine/ADMA	320.9 ± 97.0	316.5 ± 103.2	462.8 ± 31.3
Arginine/ornithine	1.8 ± 1.0	1.8 ± 0.9	2.7 ± 0.4
GABR	1.3 ± 0.6	1.3 ± 0.6	2.0 ± 0.3

Data are expressed as mean ± standard deviation unless otherwise specified. Abbreviations: ADMA, asymmetric dimethylarginine; BMI, body mass index; CRP, C-reactive protein; GABR, global arginine bioavailability ratio; IQR, interquartile range; MMA, monomethyl-l-arginine; SDMA, symmetric dimethylarginine.

**Table 2:** Changes in Serum l-Arginine Metabolites and l-Arginine Metabolism Indices after 28-day of Supplementation with l-Arginine Plus Vitamin C or Placebo in Adults with Long COVID.

Characteristic	l-Arginine + Vitamin C (*n* = 23)	Placebo (*n* = 23)	*p*
l-arginine, µM	67.8 ± 90.6	5.3 ± 81.7	0.02
Citrulline, µM	4.0 ± 10.2	2.7 ± 9.2	0.65
Ornithine, µM	30.9 ± 53.0	8.8 ± 44.8	0.13
ADMA, µM	0.07 ± 0.10	0.04 ± 0.14	0.56
MMA, µM	0.09 ± 0.17	0.03 ± 0.15	0.19
SDMA, µM	0.02 ± 0.04	0.00 ± 0.05	0.34
Arginine/ADMA	0.07 ± 0.14	−0.01 ± 0.14	0.05
Arginine/ornithine	0.03 ± 0.17	−0.02 ± 0.19	0.32
GABR	0.01 ± 0.19	−0.03 ± 0.21	0.47

Abbreviations: ADMA, asymmetric dimethylarginine; MMA, monomethyl-l-arginine; SDMA, symmetric dimethylarginine. Data for l-arginine, citrulline, ornithine, ADMA, MMA, and SDMA are expressed as deltas (values at 28 days of intervention minus baseline values) and are reported as mean ± standard deviation. Data for l-arginine-derived indices are expressed as base 10 logarithms of values at 28 days divided by values at baseline.

## Data Availability

The data presented in this study are available from the corresponding author upon reasonable request pending approval by the Gemelli against COVID Scientific Committee.
